# Enfermedades infecciosas respiratorias en pacientes de unidades de cuidados intensivos en dos hospitales de Guayaquil, Ecuador, 2024

**DOI:** 10.7705/biomedica.8220

**Published:** 2026-04-28

**Authors:** Francisco Sánchez-Amador, Miguel Merejildo, Luis Solórzano, César Bedoya, Fred Luzuriaga, Joselyne Alvarado, Marcela Miranda, Xavier Alvarado

**Affiliations:** 1 Laboratorio de Biología Molecular, Hospital Luis Vernaza, Guayaquil, Ecuador Laboratorio de Biología Molecular Hospital Luis Vernaza Guayaquil Ecuador; 2 Laboratorio de Microbiología, Hospital Luis Vernaza, Guayaquil, Ecuador Laboratorio de Microbiología Hospital Luis Vernaza Guayaquil Ecuador; 3 Centro de Referencia Nacional de Parasitología, Instituto Nacional en Investigación de Salud Pública “Leopolodo Izquieta Pérez”, Guayaquil, Ecuador Centro de Referencia Nacional de Parasitología Instituto Nacional en Investigación de Salud Pública “Leopolodo Izquieta Pérez” Guayaquil Ecuador; Laboratorio Clínico, Hospital Luis Vernaza, Guayaquil, Ecuador Laboratorio Clínico Hospital Luis Vernaza Guayaquil Ecuador; Escuela de Posgrado en Salud, UEES, Guayaquil, Ecuador Escuela de Posgrado en Salud, UEES Guayaquil Ecuador; Laboratorio de Biología Molecular, Hospital Roberto Gilbert, Guayaquil, Ecuador Laboratorio de Biología Molecular Hospital Roberto Gilbert Guayaquil Ecuador; Laboratorio de Microbiología, Hospital Roberto Gilbert, Guayaquil, Ecuador Laboratorio de Microbiología Hospital Roberto Gilbert Guayaquil Ecuador

**Keywords:** enfermedades respiratorias, enfermedades transmisibles, coinfección, indicadores de morbimortalidad, neumonía, Respiratory tract diseases, communicable diseases, coinfection, morbidity and mortality, pneumonia.

## Abstract

**Introducción.:**

Las infecciones virales, bacterianas y fúngicas constituyen un importante problema de salud, asociado a un incremento significativo de la morbimortalidad de las enfermedades respiratorias.

**Objetivo.:**

Caracterizar las infecciones respiratorias y sus agentes etiológicos en pacientes de los dos hospitales de tercer nivel de Guayaquil (Ecuador) durante los primeros seis meses del 2024.

**Materiales y métodos.:**

Estudio observacional, transversal y prospectivo de dos hospitales, entre el 1° de enero y el 30 de junio del 2024. Se recopilaron los datos demográficos, clínicos y microbiológicos (fechas, edad, sexo, tipo de muestra, resultados y diagnósticos), los cuales fueron analizados mediante el programa estadístico IBM SPSS™ Statistics 27.0.

**Resultados.:**

De los 56 pacientes incluidos, 35 presentaron infecciones respiratorias, con predominio del sexo femenino y con una edad promedio de 49 años. Se identificaron los siguientes agentes: *rinovirus-enterovirus, virus de la influenza A, coronavirus, virus de la parainfluenza y virus respiratorio sincitial*. Entre las bacterias más frecuentes se encontraron: *Klebsiella pneumoniae, Acinetobacter baumannii, Staphylococcus aureus, Pseudomonas aeruginosa, Haemophilus influenzae, Enterobacter cloacae, Escherichia coli, Proteus spp., Stenotrophomonas maltophilia, Serratia marcescens, Streptococcus agalactiae* y *Streptococcus pneumoniae*. Asimismo, se detectaron genes de resistencia de las bacterias como *CTX-M, KPC, mecA/mecC y MREJ, NDM, Oxa48-like* y *VIM*. En el caso de infecciones fúngicas, se aislaron *Candida albicans y C. glabrata*. La mayoría de los pacientes con enfermedad respiratoria evolucionaron a neumonía intrahospitalaria.

**Conclusión.:**

Durante el período estudiado, las infecciones respiratorias en las unidades de cuidados intensivos se asociaron principalmente con bacterias patógenas, destacándose la gran frecuencia de neumonías intrahospitalarias y la detección de genes de resistencia antimicrobiana.

## Abstract (en)

**Introduction.:**

Viral, bacterial, and fungal infections represent a major health concern, contributing significantly to increased morbidity and mortality in respiratory diseases.

**Objective.:**

To characterize respiratory infections and their etiological agents in patients admitted to intensive care units during the first six months of 2024.

**Materials and methods.:**

An observational, cross-sectional, prospective study was conducted in two hospitals between January 1^st^. and June 30^th^., 2024. Clinical and microbiological data were collected (dates, age, sex, type of sample, results, and diagnoses) and analyzed using IBM SPSS™ Statistics 27.0.

**Results.:**

Of the 56 patients admitted, 35 presented respiratory infections, predominantly in females, with a mean age of 49 years. The following agents were identified: rhinovirus/ enterovirus, influenza A, coronavirus, parainfluenza, and respiratory syncytial virus. Among the most frequent bacteria found were: *Klebsiella pneumoniae, Acinetobacter baumannii, Staphylococcus aureus, Pseudomonas aeruginosa, Haemophilus influenzae, Enterobacter cloacae, Escherichia coli, Proteus* spp*., Stenotrophomonas maltophilia, Serratia marcescens, Streptococcus agalactiae* and *S. pneumoniae*. Bacterial resistance genes such as *CTX-M*, *KPC*, *mecA/mecC*, and *MREJ*, *NDM*, *Oxa48-like*, and *VIM* were also detected. In the case of fungal infections, *Candida albicans* and *Candida glabrata* were isolated. Most patients with respiratory illness progressed to hospital-acquired pneumonia.

**Conclusion.:**

During the study period, respiratory infections in the intensive care units were mainly associated with bacterial pathogens, with a high frequency of hospital-acquired pneumonia and the detection of antimicrobial resistance genes.

La presencia de coinfecciones y sobreinfecciones bacterianas o fúngicas constituye un factor determinante en el incremento de la morbimortalidad asociada con las infecciones respiratorias virales ^1^. Ecuador no es ajeno a esta problemática.

En Quito, durante el periodo 2017-2018, los siguientes hospitales notificaron múltiples casos de pacientes con choque séptico: Hospital Carlos Andrade Marín, Hospital Eugenio Espejo, Hospital General Docente de Calderón y la Novaclínica Santa Cecilia.

Los principales agentes aislados fueron *Klebsiella pneumoniae* (31/88, 35,22 %), *Pseudomonas aeruginosa* (29/88, 32,95 %) y *Acinetobacter baumannii* (23/88, 24,14 %) ^2^. Asimismo, se reportaron 46 casos (56 %) de fungemias por *Candida* spp*.,* de un total de 82 pacientes que se encontraban en la unidad de cuidados intensivos. De estos pacientes, 54/82 (65,85 %) eran hombres y 28/82 (34,14 %) mujeres, con una edad media de 48 años y una estancia hospitalaria promedio de 21 días previos al diagnóstico clínico ^3^.

En Guayaquil, en el 2018, el Hospital Ceibos Norte informó que, de 80 pacientes, la neumonía asociada con asistencia respiratoria mecánica fue la principal infección intrahospitalaria (43,8 %; n = 35), seguida de la infección relacionada con catéteres intravasculares (25 %; n = 20). Los microorganismos más frecuentes fueron *K. pneumoniae* (50 %; n = 40), *A. baumannii* (25 %; n = 20), *Staphylococcus epidermidis* (18,8 %; n = 15) y *P. aeruginosa* (6,25 %; n = 5) ^4^.

En Machala, en el 2019, en el Instituto Ecuatoriano de Seguridad Social se registraron 4/56 (7,19 %) casos de neumonía asociada a la atención en salud, y *Escherichia coli* (13/30, 43,3 %) y *K. pneumoniae* (6/20, 20 %) fueron los agentes patógenos predominantes ^5^.

En el Hospital General Homero Castanier Crespo de Azogues (Cañar), y durante 2020-2021, de 50 pacientes, se reportaron 45 casos (90 %) con *K. pneumoniae*, un caso (2 %) con *Klebsiella oxytoca*, un caso (2 %) con *E. coli y* un caso (2 %) con *P. aeruginosa* en los pacientes de la unidad de cuidados intensivos ^6^.

En este contexto, resulta fundamental adelantar estudios epidemiológicos que identifiquen y caractericen los diferentes agentes infecciosos presentes en los pacientes críticos, ya sea en casos de enfermedades respiratorias o como infecciones intrahospitalarias. Este tipo de estudios contribuyen a mejorar las medidas preventivas, optimizar los cuidados paliativos, fortalecer el conocimiento clínico y establecer pautas terapéuticas más efectivas.

Los pacientes hospitalizados en las unidades de cuidados intensivos presentan una amplia variedad de agentes microbiológicos asociados con infecciones respiratorias como las neumonías intrahospitalarias.

Así pues, el propósito de la presente investigación fue caracterizar la epidemiología de las diferentes especies de virus, bacterias y hongos de los pacientes con neumonía, quienes presentan una mayor carga de factores de riesgo.

Este estudio tuvo en cuenta los reportes de los primeros seis meses del 2024, de dos hospitales de Guayaquil (Ecuador).

## Materiales y métodos

Se llevó a cabo un estudio observacional, transversal y prospectivo basado en los resultados obtenidos de las muestras procesadas en el laboratorio central del Hospital Luis Vernaza y en el del Hospital Alfredo Paulson. Se incluyeron 56 pacientes hospitalizados en las diferentes unidades de cuidados intensivos entre el 1° de enero y el 30 de junio de 2024, con diagnósticos presuntivos o definitivos de enfermedades respiratorias.

En este estudio se incluyeron:

- Todas las muestras biológicas respiratorias: lavado broncoalveolar, secreción endotraqueal, esputo, hisopado nasal, líquido pleural, aspirado o secreción traqueal, y biopsias de tejidos del aparato respiratorio (laringe, tráquea, pulmón), obtenidos por el personal médico de las unidades de cuidados intensivos.
- Todas las muestras biológicas con órdenes médicas emitidas por médicos del Hospital Luis Vernaza y del Hospital Alfredo Paulson, para: cultivos de tipo selectivos, bacilo de Koch, de hongos, tinción de Ziehl- Neelsen y tinción con tinta china (en el laboratorio de microbiología). Además, perfil de infecciones asociadas a sepsis por ADN-PCR-RT en pruebas de reacción en cadena de la polimerasa (en el laboratorio de biología molecular).
- Todas las muestras obtenidas por el personal médico o de terapia respiratoria del Hospital Luis Vernaza y del Hospital Alfredo Paulson, según los procedimientos empleados actualmente.
- Todos los pacientes hospitalizados sin importar la edad, el sexo ni su condición de ingreso Hospital Luis Vernaza y del Hospital Alfredo Paulson.
- Todos los pacientes o familiares de quienes se hubiera obtenido, de manera voluntaria, el consentimiento informado.

Se excluyeron:

- Muestras biológicas de otros órganos (aparato digestivo, aparato urinario, piel, sistema nervioso central o corazón) de pacientes con enfermedades respiratorias.
- Todos los pacientes con diagnóstico de otro tipo de enfermedad no relacionada con el aparato respiratorio.
- Todas las muestras biológicas obtenidas por personal médico de otras instituciones de salud e ingresadas al sistema de manera ambulatoria.
- Todos los pacientes de quienes no se tenía ningún tipo de información durante su permanencia en el Hospital Luis Vernaza y el Hospital Alfredo Paulson.
- Todos los pacientes que no fueron hospitalizados en las unidades de cuidados intensivos del Hospital Luis Vernaza y del Hospital Alfredo Paulson.

### Recopilación y procesamiento de la información

Tres unidades de cuidados intensivos del Hospital Luis Vernaza fueron trasladadas a las nuevas instalaciones del Hospital Alfredo Paulson, que empezaron a operar el 31 de diciembre del 2023.

Los datos demográficos y clínicos, como edad, sexo, enfermedad subyacente y esquemas antimicrobianos, se recopilaron del sistema informático de los respectivos hospitales (SERMIS®).

Se ordenaron cultivos bacterianos y fúngicos como parte de las pruebas microbiológicas estándar en el Laboratorio de Microbiología, según los procedimientos operativos ya establecidos en las guías internacionales ^7^^-^^10^. Las muestras respiratorias de lavados broncoalveolares, de líquido pleural, de pleura parietal y de esputo se procesaron mediante un método de cultivo semicuantitativo. Todas las muestras se cultivaron en agar chocolate, agar sangre, agar MacConkey y agar dextrosa Sabouraud. Se examinó el crecimiento de todas las placas de cultivo después de 24 y 48 horas ^9^^,^^10^.

Los cultivos se consideraron positivos para un agente patógeno respiratorio si se detectaban > 10^-1^ UFC/ml. Las colonias aisladas de bacterias o levaduras se identificaron mediante pruebas bioquímicas estándar (por ejemplo, catalasa, coagulasa, oxidasa, hierro de triple azúcar, citrato, ureasa, motilidad, descarboxilación de ornitina y lisina, y prueba del tubo germinativo) ^9^^,^^10^.

Las pruebas de sensibilidad a los antimicrobianos se practicaron mediante difusión en disco o microdilución en caldo, de acuerdo con las directrices del *Clinical and Laboratory Standards Institute (*CLSI,) y con los procedimientos operativos estándar del laboratorio.

El cribado de resistencia a los antimicrobianos se hizo según lo recomendado por el CLSI para el agente patógeno específico (difusión en disco de cefoxitina de 30 pg para la resistencia a la meticilina en estafilococos, prueba combinada de difusión en disco para las betalactamasas de espectro extendido y método de inactivación de carbapenémicos para carbapenemasas para bacilos Gram negativos). Cabe aclarar que solo se dispuso de los datos sobre etiología bacteriana y fúngica.

De la misma forma, se emplearon pruebas de sensibilidad para los respectivos antifúngicos (fluconazol, voriconazol, caspofungina y anfotericina B) ^8^^-^^10^.

Además, se practicaron simultáneamente pruebas de reacción de cadena polimerasa en tiempo real. Se utilizó el panel Pneumonia, versión 2, BIOFIRE^TM^ (termociclador) ^11^.

Los datos se analizaron con el *software* IBM SPSS™ Statistics 27.0. Los datos cuantitativos se expresaron como media y desviación estándar; los cualitativos se describieron como número y porcentaje ^8^.

### Consideraciones éticas

El 15 de septiembre del 2023 se obtuvo la aprobación del protocolo de investigación del Comité de Ética del Hospital Luis Vernaza, con código 13-EO-CEISH-HLV-2023. Debido a la naturaleza de la investigación y a que los procedimientos utilizados en este estudio no son invasivos, no existió riesgo físico asociado. Se elaboró un formulario de consentimiento informado para que los pacientes o los familiares directos pudieran aprobar o rechazar el uso de la información de los resultados de laboratorio, garantizándoles la confidencialidad de estos.

## Resultados

Durante enero, febrero y junio del 2024, se registraron de 3 a 5 casos positivos por mes. En cambio, en marzo, abril y mayo se observaron de 7 a 8 casos positivos de manera consecutiva cada mes. En el transcurso de los seis meses estudiados, se identificaron 35 casos (15 hombres y 20 mujeres) positivos para algún agente patógeno y 21 casos (8 hombres y 13 mujeres) negativos. La edad promedio de los pacientes fue de 49 años.

Entre los pacientes con infecciones virales detectadas mediante el panel de neumonía por biología molecular, se reportaron 4 (7 %) casos de *rinovirus-enterovirus* humanos y 1 (1,7 %) caso de cada uno de los siguientes virus: virus de influenza A, coronavirus HKU1, virus de parainfluenza y virus respiratorio sincitial (cuadro 1).

**Cuadro 1:** Aislamientos de virus obtenidos a partir de muestras respiratorias (N = 56)

Virus	n	Muestras (%)	Muestras positivas para virus (%)
Adenovirus	0	0,00	0,00
Coronavirus HKU1	1	1,79	14,29
Metapneumovirus	0	0,00	0,00
Rhinovirus/enterovirus	4	7,14	57,14
Influenza A	1	1,79	14,29
Influenza B	0	0,00	0,00
Parainfluenza	1	1,79	14,29
Sincitial respiratorio	1	1,79	14,29
Total de pacientes positivos	7	12,50	100

Se registraron 19 casos con coinfección bacteriana, 7 casos con etiología viral exclusiva, 6 casos con una única bacteria, 4 casos con coinfección viral y bacteriana, 5 casos con infección fúngica y 1 caso con coinfección por hongo y bacteria.

De los 56 pacientes hospitalizados, 29 (51,8 %) presentaron resultados negativos para infecciones bacterianas, mientras que en 27 (48,2 %), se detectó más de un agente patógeno. De las muestras positivas, 8 correspondieron a infecciones monobacterianas y 19 a infecciones polibacterianas (con dos o más bacterias). Además, de 27 muestras, en 13 (23,2 %) se observaron mecanismos de resistencia y, en 5 (8,9 %), estos fueron múltiples.

Los agentes etiológicos fueron aislados mediante paneles de neumonía por PCR-RTy cultivos, distribuyéndose de la siguiente manera: *K. pneumoniae,* 15 (26,7 %) casos; *A. baumannii',* 13 (23,2 %) casos; *Staphylococcus aureus*, 8 (14,2 %) casos; *P. aeruginosa*, 7 (12,5 %) casos; *Haemophilus influenzae y Enterobacter cloacae*, 4 (7,1 %) casos cada uno; *E. coli*, *Proteus spp.* y *Stenotrophomonas maltophilia*, 3 (5,36 %) casos cada uno; y *Serratia marcescens*, *Streptococcus agalactiae* y *Streptococcus pneumoniae*, 1 (1,79 %) caso cada uno (cuadro 2).

**Cuadro 2:** Aislamientos de bacterias obtenidos a partir de muestras respiratorias (N = 56)

Bacteria	n	Muestras (%)	Muestras positivas para bacterias (%)
*Acinetobacter baumannii*	13	23,21	48,15
*Enterobacter cloacae*	4	7,14	14,81
*Escherichia coli*	3	5,36	11,11
*Haemophilus influenzae*	4	7,14	14,81
*Klebsiella aerogenes*	0	0,00	0,00
*Klebsiella oxytoca*	0	0,00	0,00
*Klebsiella pneumoniae*	15	26,79	55,56
*Moraxella catarrhalis*	0	0,00	0,00
*Proteus* spp*.*	3	5,36	11,11
*Pseudomonas aeruginosa*	7	12,50	25,93
*Serratia marcescens*	1	1,79	3,70
*Staphylococcus aureus*	8	14,29	29,63
*Streptococcus agalactiae*	1	1,79	3,70
*Streptococcus pneumoniae*	1	1,79	3,70
*Streptococcus pyogenes*	0	0,00	0,00
*Stenotrophomonas maltophilia*	3	5,36	11,11
Total de pacientes positivos	27	48,2	100

En cuanto a los mecanismos de resistencia bacteriana, el más frecuente fue la producción de betalactamasas, destacándose las de espectro extendido (BLEE) como la *CTX-M*, presente en 9 (16 %) muestras de las infecciones respiratorias, seguida de la *KPC y mecA/mecCy* la *MREJ* en 4 (7,1 %) muestras, y *NDM*, *VIM y* de tipo *OxA-48* en 1 (1,8 %) muestra cada uno.

En los 7 (12,5 %) pacientes con infecciones por hongos patógenos, se aisló *Candida albicans* en 6 (85,7 %) casos, de los cuales 2 (28,6 %) presentaron resistencia a fluconazol, y *Candida glabrata* en 1 (14,3 %) caso, también resistente a fluconazol (cuadro 3).

**Cuadro 3:** Aislamientos de agentes micológicos obtenidos a partir de muestras respiratorias (N = 56)

Hongo	n	Muestras (%)	Muestras positivas para hongos (%)
*Candida albicans*	6	10,71	85,71
*Candida glabrata*	1	1,79	14,29
*Candida albicans* resistentes a fluconazol	2	3,57	28,57
*Candida glabrata* resistente a fluconazol	1	1,79	14,29

De las 56 muestras estudiadas mediante cultivos y PCR-RT, 48 correspondieron a lavados bronqueoalveolares, de los cuales 33 resultaron positivos para algún agente patógeno y 15 negativos. De las 8 muestras restantes, 4 de líquido pleural y 2 de pleura parietal fueron negativas, y 2 de esputo resultaron positivas para algún agente infeccioso.

Con respecto al diagnóstico de ingreso en las unidades de cuidados intensivos, las causas más frecuentes fueron: enfermedades respiratorias en 18 (32 %) casos; enfermedades gastrointestinales en 13 (23 %) casos; enfermedades neurológicas en 10 (18 %); politraumatismos en 7 (13 %); enfermedades hematológicas en 4 (7 %) casos; enfermedades cardiovasculares en 3 (5 %) e insuficiencia renal en uno (2 %).

Los diagnósticos definitivos reportados por las diferentes unidades de cuidados intensivos, a partir de los 56 casos seleccionados, fueron los siguientes: 42 neumonías intrahospitalarias en pacientes remitidos de otros hospitales y posquirúrgicos, 6 derrames pleurales, 3 neumonías adquiridas en la comunidad, 2 insuficiencias respiratorias agudas, 2 septicemias (cavidad cardiotorácica) y 1 caso de tumor benigno del aparato respiratorio (cuadro 4).

**Cuadro 4:** Diagnósticos definitivos reportados en pacientes hospitalizados en las unidades de cuidados intensivos del Hospital Luis Vernaza y el Hospital Alfredo Paulson durante enero y junio del 2024 (N = 56)

Diagnósticos definitivos	n
Neumonía intrahospitalaria	42
Neumonía adquirida en la comunidad	3
Insuficiencia respiratoria aguda	2
Septicemias	2
Derrame pleural	6
Tumor	1

## Discusión

En el presente estudio, se caracterizaron las infecciones respiratorias de las unidades de cuidados intensivos de dos hospitales de Guayaquil durante el primer semestre del 2024, con una prevalencia del 62,5 % (35/56) de infecciones confirmadas microbiológicamente, comparable con los reportes internacionales que documentan tasas del 62 % (45/98) en centros terciarios utilizando paneles moleculares ^8^^,^^11^.

Es importante considerar que en el 2024 se reportó una humedad absoluta alta en Guayaquil, especialmente durante la temporada de lluvias de enero a mayo, con promedios que se encontraban entre el 80 y el 88 %. La humedad relativa se mantuvo en aumento durante la mayor parte del año, con valores mayores del 60 % ^12^.

La edad promedio de 49 años se alinea con los estudios mundiales en los cuales los pacientes críticos se agrupan entre la quinta y la sexta décadas de la vida ^13^^,^^14^. El predominio femenino observado (57,1 %) difiere de la tendencia internacional que reporta ligera prevalencia masculina ^15^.

El virus màs prevalente fue el rinovirus-enterovirus (57 % de muestras virales), seguido por el virus de la influenza A, el coronavirus HKU1, el virus de la parainfluenza y el virus sincitial respiratorio. Estos hallazgos coinciden con los de los estudios internacionales ^16^^,^^17^.

Las coinfecciones bacterianas (54,3 %) superaron a las de los reportes internacionales del 42 al 45 % ^18^^,^^19^, mientras que las coinfecciones virales-bacterianas (11,4 %) presentaron una particular complejidad clínica. Las infecciones virales, aunque menos frecuentes (12,5 %), desempeñan un papel crucial como desencadenantes de superinfecciones bacterianas mediante daño epitelial, alteración ciliar y modulación inmunitaria ^1^.

El perfil microbiològico reveló características distintivas: *Klebsiella pneumoniae* (26,79 %) y *A. baumannii* (23,21 %) predominaron, seguidas por *S. aureus* (14,29 %) y *P. aeruginosa* (12,5 %). Este patrón difiere de los estudios norteamericanos, donde *S. aureus* lidera con 22 % (16). Sin embargo, los hallazgos concuerdan con los reportes asiàticos que reportaron *A. baumannii* (5/67, 7,5 %) y *K. pneumoniae* (10/67, 14,9 %) (20) Estos resultados también van en línea con los estudios latinoamericanos que reportaron *A. baumanii* (7/253, 3 %) y *K. pneumoniae* (33/253, 13 %) como las bacterias patógenas prevalentes ^18^.

La detección de genes de resistencia antimicrobiana en 23,2 % de las muestras constituye un hallazgo alarmante. *CTX-M* (16 %) fue el más frecuente, seguido por *KPCy mecA/mecC*(7,1 % cada uno). Estas tasas son superiores a las de los reportes internacionales que documentan del 13,3 al 22 % de genes de resistencia ^21^^,^^22^, lo cual refleja la creciente amenaza de multirresistencia en las unidades de cuidados intensivos latinoamericanas.

Las carbapenemasas NDM, VIM y OXA-48-like presentaron una frecuencia de 1,8 % cada una. La presencia de carbapenemasas en el 10,7 % de las muestras positivas, limita drásticamente las opciones terapéuticas y subraya la urgencia de implementar programas sólidos de vigilancia antimicrobiana ^4^^,^^6^.

Las infecciones fúngicas fueron predominantemente por *C. albicans* (33/90, 36,7 %), y con menor frecuencia, *C. glabrata* (19/90, 21,1 %) ^3^. En el Hospital del Día del IESS se reportaron 132/169 (78 %) pacientes con *C. albicans*; 39/169 (23 %) presentaron resistencia al fluconazol ^23^.

El FilmArray™ Pneumonia Panel emergió como herramienta fundamental en la vigilancia epidemiológica, detectándose 33 blancos (18 bacterias, 8 virus, 7 genes de resistencia) en aproximadamente una hora *vs*. 44 a 56 horas por medio de los métodos convencionales ^11^.

La sensibilidad del 94 al 96 % y la especificidad del 95 al 98,3 % ^8^^,^^11^, junto con la concordancia del 99,2 % para virus y 96,8 % para bacterias con métodos estándar ^16^^,^^19^ demuestran su utilidad diagnóstica. La capacidad de semicuantificación bacteriana permite diferenciar una colonización de una infección verdadera, aspecto crítico en la interpretación de los cultivos respiratorios ^11^^,^^19^.

El impacto del FilmArray™ en la vigilancia de la resistencia antibacteriana es significativo. En los estudios controlados se ha demostrado que las pruebas moleculares del tipo de FilmArray™ permiten una desescalada segura de antibióticos y reducen el uso innecesario de los de amplio espectro ^11^^,^^21^.

La detección temprana de los genes de resistencia permite ajustar inmediatamente la terapia empírica, evitando la administración de antibióticos inefectivos y reduciendo la presión selectiva que favorece la aparición de resistencia ^18^^,^^21^^,^^24^. Los análisis de costo-beneficio demuestran que el FilmArray™, aunque tiene mayor costo inicial, resulta en ahorros netos al optimizar el tratamiento antimicrobiano y reducir la estancia hospitalaria ^19^^,^^21^.

La detección viral rápida facilita la implementación oportuna de las precauciones de aislamiento, reduciendo la transmisión intrahospitalaria. Se ha documentado que los paneles moleculares disminuyen el tiempo de implementación de las medidas de aislamiento, pasando de 24-48 horas a menos de 4 horas ^8^^,^^16^^,^^19^. Esto representa una contribución significativa al control de brotes hospitalarios.

En los estudios en los que se combinaron la PCR-RT y los cultivos para estudiar muestras respiratorias -1035 esputos, 187 lavados broncoalveolares, 2 biopsias de pulmón y 146 líquidos pleurales-, se detectaron 80 % de muestras positivas, resultados similares a los del presente estudio de seis meses, que presentó un 63 % de positividad para distintos agentes patógenos ^25^. De igual forma, predominaron tanto muestras positivas como negativas de lavados broncoalveolares, obtenidas por fibrobroncoscopia en pacientes de unidades de cuidados intensivos.

Se debe tener en cuenta que los cambios bruscos de temperatura, comunes en los climas húmedos, debilitan el sistema inmunológico y agravan las condiciones respiratorias preexistentes, induciendo a infecciones respiratorias con insuficiencia respiratoria, como bronquitis y neumonías ^26^. También, cabe resaltar que detectamos mayormente infecciones respiratorias en los primeros meses del año.

En estudios epidemiológicos de varios países, se describen las causas más comunes según el diagnóstico de ingreso a la unidad de cuidados intensivos: enfermedades respiratorias (46,8 %) ^19^, enfermedades neurológicas (24,5 %) ^27^, traumatismos (14 %), enfermedades cardiológicas (22 %) ^20^, insuficiencia renal (9,6 %) ^28^, enfermedades gastrointestinales (6,8 %) ^20^ y enfermedades hematológicas (1,8 %) ^28^.

La elevada frecuencia de neumonía intrahospitalaria (75 %) superó la de reportes internacionales que indicaban prevalencias del 12 al 52,5 % ^27^, reflejando múltiples factores de riesgo: asistencia respiratoria mecánica prolongada, estancias extensas, enfermedades graves y uso previo de antibióticos.

La mortalidad por neumonía intrahospitalaria en las unidades de cuidados intensivos oscila mundialmente entre el 25 y el 72,6 %, con tasas atribuibles del 33 al 50 % ^29^^,^^30^. Los factores asociados con mayor mortalidad incluyen la presencia de organismos multirresistentes, especialmente *A. baumannii* y *P. aeruginosa*, y las demoras en administrar los antibióticos apropiados ^24^^,^^27^.

Las limitaciones de este estudio incluyen el tamaño muestral (n = 56), un periodo limitado (seis meses) y la ausencia de seguimiento a largo plazo. Una limitación técnica del FilmArray™ es la exclusión de blancos fúngicos (excepto *Pneumocystis jirovecii y S. maltophilia)*, detectada en 5,36 % de las muestras mediante cultivos convencionales. Esto subraya la necesidad de mantener los cultivos tradicionales complementarios ^11^^,^^24^.

En conclusión, este estudio documenta la importante carga de infecciones respiratorias presente en las unidades de cuidados intensivos, con predominio de las bacterias Gram negativas multirresistentes, patrón consistente con el de otros estudios llevados a cabo en Latinoamérica y Asia.

La frecuencia de resistencia antimicrobiana (23,2 %) y de neumonías intrahospitalarias (75 %), subraya la urgencia de fortalecer las estrategias de prevención y control. La implementación sistemática de las tecnologías moleculares, integradas en algoritmos clínicos basados en la evidencia y en programas de vigilancia, representa una estrategia prometedora para reducir la morbimortalidad en las unidades de cuidados intensivos, sin importar la estación del año.

En Ecuador, donde la resistencia antimicrobiana es creciente, la adopción de estas tecnologías debe acompañarse de directrices que fortalezcan la vigilancia epidemiológica a nivel local y nacional ^29^^,^^31^.

## References

[1] Nebreda-Mayoral T, Miguel-Gómez A, March-Rosselló A, Puente-Fuertes L, Cantón-Benito E, Martínez-García M (2022). Infección bacteriana/fúngica en pacientes con COVID-19 ingresados en un hospital de tercer nivel de Castilla y León, España. Enferm Infecc Microbiol Clin..

[2] González D. (2020). Efectividad clínica de esquemas de tratamiento antibiótico para cepas de Gram negativos productores de carbapenemasas aisladas en pacientes con sepsis choque séptico, hospitalizados en unidades de cuidados intensivos de la ciudad de Quito (tesis).

[3] Rivadeneira Negrete AD. (2019). Características clínico epidemiológicas de fungemias en el Hospital de Especialidades “Carlos Andrade Marín” 2016-2018 (tesis).

[4] Neira J, Espinoza C, Mejía C, Mesías J, Llerena G, Toapanta L (2021). Microorganismos multirresistentes en la unidad de cuidados intensivos del Hospital General del Norte Los Ceibos.

[5] Lam-Vivanco A, Sotomayor-Preciado A, Santos-Luna J, Espinoza-Carrión F.

[6] Ochoa C, Ordóñez D, Alvarado M, Ramírez A. (2022). Infección bacteriana asociada a ventilación mecánica en pacientes de UCI-COVID-19 del Hospital Homero Castanier Crespo. Revista Ecuatoriana de Ciencia. Tecnología e Innovación en Salud Pública.

[7] Zambrano S, Castro J. (2023). Epidemiología, factores de riesgo y especies de cándidas en pacientes de la unidad de cuidados intensivos. Polo del Conocimiento.

[8] El-Nawawy A, Antonios A, Tawfik E, Meheissen A. (2022). Comparison of a point-of-care filmarray test to standard-of-care microbiology test in diagnosis of healthcare associated infections in a tertiary care pediatric intensive care unit. Antibiotics.

[9] 9. Tille B, Scott’s. Diagnostic Microbiology. 14th edition. St Louis MO, USA: Mosby Elsevier; 2017.

[10] Clinical and Laboratory Standards Institute Performance standards for antimicrobial susceptibility testing.

[11] Buchan W, Windham S, Balada-Llasat M, Leber A, Harrington A, Relich R. (2020). Practical comparison of the BioFire FilmArray pneumonia panel to routine diagnostic methods and potential impact on antimicrobial stewardship in adult hospitalized patients with lower respiratory tract infections. J Clin Microbio.

[12] Spark. Weather Datos históricos meteorológicos de 2024 en Guayaquil.

[13] Intensive Care National Audit & Research Centre-ICNARC (2022). Key statistics from the case mix programme 2021-22.

[14] Vincent JL, Rello J, Marshall J, Silva E, Anzueto A, Martin CD (2009). International study of the prevalence and outcomes of infection in intensive care units. JAMA.

[15] Johnson AE, Pollard TJ, Shen L, Lehman LW, Feng M, Ghassemi M (2016). MIMIC-III, a freely accessible critical care database. Sci Data.

[16] Webber M, Wallace A, Burnham D, Anderson W. (2020). Evaluation of the BioFireFilmArray pneumonia panel for detection of viral and bacterial pathogens in lower respiratory tract specimens in the setting of a tertiary care academic medical center. J Clin Microbiol.

[17] Montiel-Jarolin D, Samudio M, Volkart K, Leguizamón R, Jarolin M, Torres E (2024). Caracterización clínico-epidemiológica agentes etiológicos de las infecciones respiratorias agudas graves en adultos en Paraguay. Med Clin Soc.

[18] Soloaga R, Barcán L, Bettiol M, Carrión N, Cornistein W, Cristofano A (2021). Estudio multicéntrico argentino sobre la utilidad del panel de neumonía de FilmArray. Act Bioquím Clín Latinoam.

[19] Escudero D, Fernández-Suarez J, Forcelledo L, Balboa S, Fernández J, Astola I (2022). Evaluation and clinical impact of BiofireFilmArray pneumonia panel plus in ICU-hospitalized COVID-19 patient. Diagnostics (Basel).

[20] Yoo Y, Seok S, Kwon A, Lee J, Jo S, Kim Y (2023). Evaluation of the BioFireFilmArray® pneumonia panel with conventional bacterial culture in conjunction with leukocyte esterase test. Diagnostics.

[21] Trejos-Valencia JC Largo-Ocampo J, Ortiz-Rojas-JH López-Vargas JA. (2025). Performance of the FilmArray® pneumonia panel compared to bacterial culture in patients who were requested both diagnostic methods. Infectio.

[22] Vásquez-Zamora G, Villalobos-Barboza K, Vergara-Espinoza A, Ventura-Flores R, Silva- Díaz H. (2020). Frecuencia y susceptibilidad antifúngica de Candida spp. (no C. albicans) aislada de pacientes de unidades de cuidados críticos de un hospital de tercer nivel del norte del Perú. Horizonte Médico (Lima).

[23] Lara J. (2019). Cepas de Candida albicans, aisladas en pacientes con diabetes mellitusy su resistencia a los antifúngicos en el Hospital del día IESS. Recimundo.

[24] Metlay P, Waterer W, Long C, Anzueto A, Brozek J, Crothers K (2019). Diagnosis and treatment of adults with community-acquired pneumonia an official clinical practice guideline of the American Thoracic Society and Infectious Diseases Society of America. Am J Respir Crit Car Med.

[25] Amaya G, Contrera M, Arrieta F, Montano A, Pírez C. (2020). Actuación de GeneXpert en el diagnóstico de tuberculosis pulmonar y extrapulmonar en la edad pediátrica. Arc Pedia Urug.

[26] Medina D. Enfermedades respiratorias: expertas U de C alerta los peligros de la alta humedad en viviendas durante el invierno.

[27] León M, Claro D, Cruz L, Vázquez J, Turro R. (2019). Microorganismos causales de neumonía asociada a la ventilación mecánica. Rev Inf Cient.

[28] Peraro-Corréa G, Fogaga-Rabito B, Matias-Ciccheto R, Aparecida-Salci M, Oliveira- Moura R, Cassia-Nogueira-Sanches R. (2024). Perfil clínico-epidemiológico de los pacientes ingresados en la unidad de cuidados intensivos de un hospital escuela. Enfermería Global.

[29] Rodríguez C, Barrios D, García A. (2019). Actualización de las infecciones respiratorias en Urgencias. Medicine (Madr).

[30] Olaechea M, Álvarez F, Beato C, Gimeno R, Gordo F, Durá R (2020). Epidemiología y pronóstico de los pacientes con antecedentes de neoplasia ingresados en las unidades de cuidados intensivos. Estudio multicéntrico observacional. Medicina Interna.

[31] Ruilova K, Velasco K, Pienda R, Salazar D. (2021). Manejo de shock séptico en unidad de cuidados intensivos. RECIMAUC.

